# *PAPPA2* mutation as a novel indicator stratifying beneficiaries of immune checkpoint inhibitors in skin cutaneous melanoma and non‐small cell lung cancer

**DOI:** 10.1111/cpr.13283

**Published:** 2022-07-10

**Authors:** Yiting Dong, Lele Zhao, Jianchun Duan, Hua Bai, Dongsheng Chen, Si Li, Yangyang Yu, Mingzhe Xiao, Qin Zhang, Qianqian Duan, Tingting Sun, Chuang Qi, Jie Wang, Zhijie Wang

**Affiliations:** ^1^ State Key Laboratory of Molecular Oncology, Department of Medical Oncology, National Cancer Center/National Clinical Research Center for Cancer/Cancer Hospital Chinese Academy of Medical Sciences and Peking Union Medical College Beijing China; ^2^ The Medical Department, Jiangsu Simcere Diagnostics Co., Ltd, Nanjing Simcere Medical Laboratory Science Co., Ltd, The State Key Laboratory of Translational Medicine and Innovative Drug Development Jiangsu Simcere Diagnostics Co., Ltd Nanjing China

## Abstract

**Background:**

Pappalysin 2 (*PAPPA2*) mutation, occurring most frequently in skin cutaneous melanoma (SKCM) and non‐small cell lung cancer (NSCLC), is found to be related to anti‐tumour immune response. However, the association between *PAPPA2* and the efficacy of immune checkpoint inhibitors (ICIs) therapy remains unknown.

**Methods:**

To analyse the performance of *PAPPA2* mutation as an indicator stratifying beneficiaries of ICIs, seven public cohorts with whole‐exome sequencing (WES) data were divided into the NSCLC set (*n* = 165) and the SKCM set (*n* = 210). For further validation, 41 NSCLC patients receiving anti‐PD‐(L)1 treatment were enrolled in China cohort (*n* = 41). The mechanism was explored based on The Cancer Genome Atlas database (*n* = 1467).

**Results:**

In the NSCLC set, patients with *PAPPA2* mutation (PAPPA2‐Mut) demonstrated a significantly superior progress free survival (PFS, hazard ratio [HR], 0.28 [95% CI, 0.14–0.53]; *p* < 0.001) and objective response rate (ORR, 77.8% vs. 23.2%; *p* < 0.001) compared to those with wide‐type *PAPPA2* (PAPPA2‐WT), consistent in the SKCM set (overall survival, HR, 0.49 [95% CI: 0.31–0.78], *p* < 0.001; ORR, 34.1% vs. 16.9%, *p* = 0.039) and China cohort. Similar results were observed in multivariable models. Accordingly, *PAPPA2* mutation exhibited superior performance in predicting ICIs efficacy compared with other published ICIs‐related gene mutations, such as *EPHA* family, *MUC16*, *LRP1B* and *TTN*, etc. In addition, combined utilization of *PAPPA2* mutation and tumour mutational burden (TMB) could expand the identification of potential responders to ICIs therapy in both NSCLC set (HR, 0.36 [95% CI: 0.23–0.57], *p* < 0.001) and SKCM set (HR, 0.51 [95% CI: 0.34–0.76], *p* < 0.001). Moreover, *PAPPA2* mutation was correlated with enhanced anti‐tumour immunity including higher activated CD4 memory T cells level, lower Treg cells level, and upregulated DNA damage repair pathways.

**Conclusions:**

Our findings indicated that *PAPPA2* mutation could serve as a novel indicator to stratify beneficiaries from ICIs therapy in NSCLC and SKCM, warranting further prospective studies.

## Graphical abstract

Flow diagram of the study. (A) Preliminary analysis. *PAPPA2* mutated most frequently in skin cutaneous melanoma (SKCM) and non‐small cell lung cancer (NSCLC) in the The Cancer Genome Atlas (TCGA) database. *PAPPA2* mutational rates in patients with objective response (CR + PR) versus without (SD + PD) were compared with other immune checkpoint inhibitors‐related gene mutations in the NSCLC and SKCM sets. (B) Biomarker development. Association between *PAPPA2* mutation and clinical outcomes has been analysed in the NSCLC set, the SKCM set and China cohort. (C) Mechanism exploring. Based on the TCGA database, the correlation of *PAPPA2* mutation with tumour mutation burden, infiltrating immune cells and DNA damage repair was explored for further immunogenicity and anti‐tumour activity mechanisms.

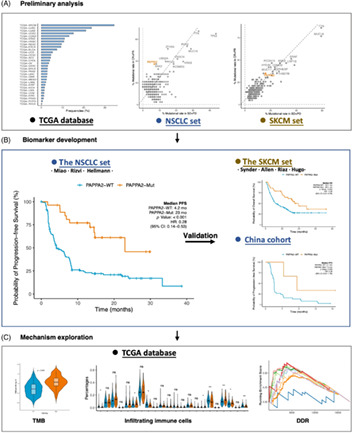

## 1 INTRODUCTION

Cancer is the leading cause of death in the world. In 2020, there were an estimated 19.3 million new cancer cases and nearly 10 million cancer deaths worldwide.^1^, ^2^ The advancement in medical technology has opened several avenues in the diagnosis and treatment of various diseases including cancer.^3^, ^4^, ^5^, ^6^

Immune checkpoint inhibitors (ICIs), targeting the programmed cell death 1 (PD‐1), programmed cell death ligand 1 (PD‐L1) or cytotoxic T lymphocyte‐associated protein 4 (CTLA‐4), have demonstrated impressive anti‐tumour efficacy in multiple cancers, in particular with non‐small cell lung cancer (NSCLC)^7^ and skin cutaneous melanoma (SKCM).^8^ However, the response rates of 10% ~ 20% in NSCLC^9^ and 30% ~ 40% in SKCM^10^ indicated that only a part of patients were therapeutic beneficiaries. Therefore, investigating predictive biomarkers of clinical outcomes from ICIs therapy is of great importance to identify the target population.^11^, ^12^

Even though the expression of PD‐L1, microsatellite instability, and tumour mutation burden (TMB) exhibited predictive utility to ICIs therapy response in some clinical practices,^13^, ^14^, ^15^ the value of single gene prediction has been widely concerned, considering its relatively efficient and cost‐effective detection property. For instance, *EPHA*,^16^ *NOTCH4*,^17^ and *LRP1B* ^18^ mutations are all independent classifiers that could stratify beneficiaries of ICIs therapy. Although these biomarkers have been verified in some clinical trials, some limitations and indeterminacies remained. Therefore, it is necessary to explore other novel biomarkers to precisely maximize the identification of potential responders to ICIs treatment.

Pappalysin2 (PAPPA2) protein, a member of the pappalysin family of metzincin metalloproteinases, has been identified as a subset of insulin growth factor (IGF)‐binding proteins.^19^, ^20^ The decreasing of free IGF‐1 levels led by dysfunction of PAPPA2 protein could result in an imbalanced growth hormone (GH)/IGF‐1 signalling pathway, which was related to DNA damage repair (DDR) pathway, immune system maintenance and anti‐tumour immune activation.^21^, ^22^, ^23^, ^24^ Meantime, another study suggested that patients with *PAPPA2* mutation (PAPPA2‐Mut) showed a prolonged survival time in lung adenocarcinoma (LUAD).^25^ However, the understanding of the contribution of *PAPPA2* mutation to the anti‐tumour immune system is still lacking and remains to be explored.

In this study, we observed that *PAPPA2* mutated most frequently in NSCLC (22.2% mutant) and SKCM (34.3% mutant) based on the The Cancer Genome Atlas (TCGA) database. Therefore, to figure out the impact of *PAPPA2* mutation on the clinical outcome of ICIs treatment, we investigated the association between *PAPPA2* mutation and clinical efficacy of ICI in several NSCLC and SKCM cohorts. The underlying mechanisms were subsequently explored based on RNA expression and whole‐genome sequencing (WES) data from the TCGA database.

## 2 METHODS

### 2.1 Cohort description and data compilation

To investigate the association between *PAPPA2* mutation and immunotherapy efficacy, seven public cohorts with WES data were collected and divided into an NSCLC set (*n* = 165) and a SKCM set (*n* = 210).

The NSCLC set (*n* = 165) was a pooled set consisting three independent cohorts (Rizvi cohort,^26^ Hellmann cohort,^27^ and Miao cohort^28^). The SKCM set (*n* = 210) was a pooled set consisting four public cohorts (Synder cohort,^29^ Allen cohort,^30^ Riaz cohort,^31^ and Hugo cohort^32^).

We also obtained TCGA data of LUAD and SKCM to explore the mechanism underlying the association between *PAPPA2* mutation and immunotherapy. The RNA sequencing (RNA‐Seq) data were retrieved from Genomic Data Commons (GDC) Data Portal (https://portal.gdc.cancer.gov/). The genomic data of WES and TMB in each TCGA tumour sample were obtained from Hoadley et al.^33^ Survival data were retrieved from UCSC Xena data portal (https://xenabrowser.net). Information regarding the neoantigen load (NAL) and CIBERSORT‐inferred values in each TCGA tumour sample was obtained from Thorsson et al.^34^

### 2.2 Patient enrollment

For further validation, we included NSCLC patients treated with anti‐PD‐(L)1 at the National Cancer Center/National Clinical Research Center for Cancer/Cancer Hospital and Chinese Academy of Medical Sciences and Peking Union Medical College (NCC) and Sun Yat‐sen University Cancer Center (SYUCC) from December 2016 to December 2018 (China cohort, *n* = 41). Eligible patients were 18 years of age or older with advanced or recurrent NSCLC diagnosed using Response Evaluation Criteria in Solid Tumours (RECIST) version 1.1 by investigator review, and an Eastern Cooperative Oncology Group performance status of 0 or 1. Key exclusion criteria included known active central nervous system metastases, diagnosis of immunodeficiency, prior immunotherapy for other diseases, autoimmune disease or active infection that required systemic therapy. WES analysis was performed on the tissue samples of all 41 patients. This study was approved by the ethics committees of the participating centres and all patients provided written informed consent.

### 2.3 Clinical outcomes

The primary clinical outcomes were progress free survival (PFS) and overall survival (OS). The secondary clinical outcomes were objective response rate (ORR), disease control rate (DCR) and durable clinical benefit (DCB). ORR and DCR were assessed using RECIST 1.1 (irRECIST for Hugo cohort, irRC for Rizvi cohort). The survival data available in the NSCLC cohorts (Rizvi, Hellmann, Miao and China cohorts) is PFS, while the survival data in common for SKCM cohorts (Synder, Allen, Riaz, and Hugo cohorts) is OS. The details of DCB definition are shown in Table S1.

### 2.4 *PAPPA2* gene mutation

Patients with nonsynonymous somatic mutations in the coding region of the *PAPPA2* gene were defined as *PAPPA2*‐mutant (PAPPA2‐Mut) and patients without were defined as *PAPPA2*‐wildtype (PAPPA2‐WT).

### 2.5 TMB data analysis

TMB for immunotherapy cohorts and TCGA datasets was defined as the total number of nonsynonymous somatic mutations and the total number of nonsynonymous somatic mutations per megabase of genome examined, respectively. The cutoff value for high and low TMB in this study was the top 25% TMB within each set.^35^, ^36^

### 2.6 DDR pathways and gene sets

The core genes associated with DDR pathways were obtained from Knijnenburg et al.^37^ The details of DDR core genes are shown in Table S2. The DDR gene sets were obtained from the Reactome Knowledgebase (https://reactome.org).^38^ The details of DDR gene sets are shown in Table S3.

### 2.7 Gene set enrichment analysis

R package DESeq2 was conducted for differential gene expression analysis.^39^ Reactome pathways analysis based on Gene set enrichment analysis (GSEA) was performed by the R package ClusterProfiler.^40^ Gene sets with an adjusted *p* value (Benjamini‐Hochberg method) lower than 0.05 were considered significantly enriched.

### 2.8 Statistical analysis

The differences in TMB, NAL, tumour‐infiltrating leukocytes and gene expressions between PAPPA2‐Mut and PAPPA2‐WT groups were examined using the Mann–Whitney *U* test. Comparisons of ORR, DCR, DCB, and PD‐L1 expression in different sets were conducted with Fisher's exact test. PFS and OS were estimated by Kaplan–Meier method, with the *p* value determined by a log‐rank test. The Cox regression was applied for univariable and multivariable survival analyses. Variables with *p* < 0.05 in the univariable regression and those which have been reported associated with the effect of ICIs were also included in multivariable Cox regression. All the statistical analyses were performed using R version 4.1.1 (https://www.r-project.org). All reported *p* values were two‐tailed, and *p* < 0.05 was considered statistically significant.

## 3 RESULTS

### 3.1 Association between *PAPPA2* mutation and the clinical benefit of ICIs therapy in the NSCLC set

**FIGURE 1:**
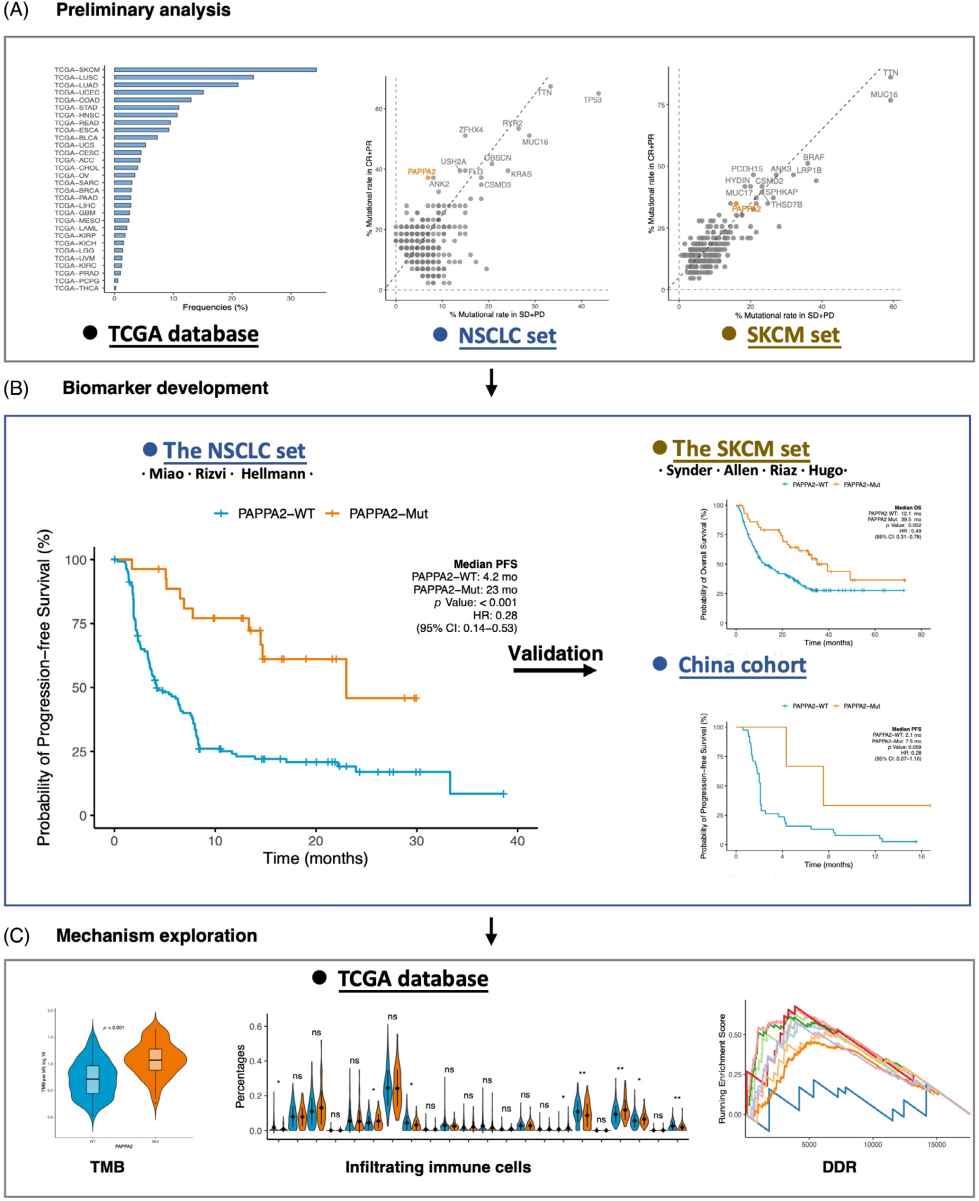
Flow diagram of the study. (A) Preliminary analysis. *PAPPA2* mutated most frequently in SKCM and NSCLC in the TCGA database. *PAPPA2* mutational rates in patients with objective response (CR + PR) versus without (SD + PD) were compared with other ICIs‐related gene mutations in the NSCLC and SKCM sets. (B) Biomarker development. Association between *PAPPA2* mutation and clinical outcomes has been analysed in the NSCLC set, the SKCM set and China cohort. (C) Mechanism exploring. Based on the TCGA database, the correlations of *PAPPA2* mutation with TMB, infiltrating immune cells and DDR were explored for further immunogenicity and anti‐tumour activity mechanisms. DDR, DNA damage repair; NSCLC, non‐small cell lung cancer; SKCM, skin cutaneous melanoma; TCGA, The Cancer Genome Atlas; TMB, tumour mutation burden

The flow diagram of this study is depicted in Figure 1. In this study, we observed that *PAPPA2* mutation was mostly enriched in patients with NSCLC and SKCM while strongly differenced in patients with objective response to ICIs versus without in the NSCLC and SKCM sets. To identify whether *PAPPA2* mutation was associated with the response to ICIs therapy, we integrated the mutational and clinical data of three NSCLC cohorts (Rizvi, Hellmann and Miao cohorts) and four SKCM cohorts (Synder, Allen, Riaz cohort and Hugo cohorts) to form the NSCLC and SKCM set, respectively. Tables S4 and S5 summarized the clinical characteristics of patients in the NSCLC set and the SKCM set, respectively.

**FIGURE 2:**
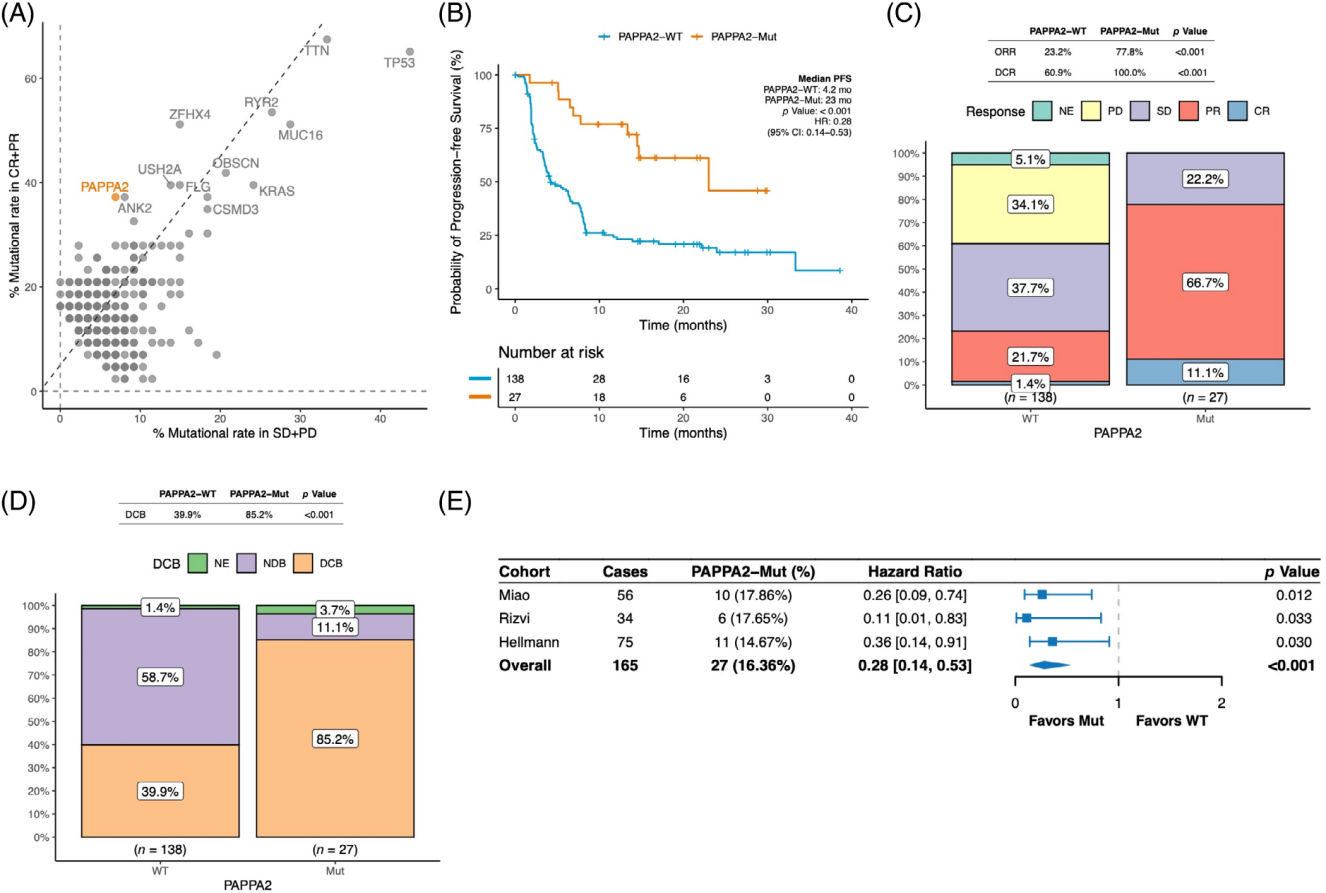
Association between *PAPPA2* mutation and clinical benefits of ICIs therapy in the NSCLC set. (A) A scatter diagram displaying the mutational rate of different ICIs‐related gene mutations in patients with objective response (CR + PR) versus without (SD + PD) in the NSCLC set. *PAPPA2* mutation (39.6% vs. 6.3%) is highlighted in orange. (B) Longer PFS observed in the PAPPA2‐Mut group compared to the PAPPA2‐MT group in the NSCLC set. (C) The response data on ORR and DCR of patients evaluated in the NSCLC set. (D) The response data on DCB of patients evaluated in the NSCLC set. (E) The prolonged PFS in the PAPPA2‐Mut group consistently observed among three cohorts included in the NSCLC set. DCB, durable clinical benefit; DCR, disease control rate; ICIs, immune checkpoint inhibitors; NSCLC, non‐small cell lung cancer; ORR, objective response rate; PFS, progress free survival

Among all candidates, *PAPPA2* mutation was discovered to be enriched in patients with objective response (39.6%) versus without (6.3%) in the NSCLC set (Figure 2A). As expected, compared to that in the PAPPA2‐WT group, longer PFS was observed in the PAPPA2‐Mut group (hazard ratio [HR], 0.26 [95% CI: 0.14–0.53]; *p* < 0.001, Figure 2B). Patients harbouring PAPPA2‐Mut had a higher ORR (77.8% vs. 23.2%; *p* < 0.001; Figure 2C) and a higher DCR (100.0% vs. 60.9%; *p* < 0.001; Figure 2C). Meanwhile, a higher DCB (85.2% vs. 39.9%; *p* < 0.001; Figure 2D) in PAPPA2‐Mut compared with those PAPPA2‐WT was confirmed. The result of prolonged PFS in PAPPA2‐Mut patients was consistently observed across all three cohorts (Rizvi, Hellmann, and Miao cohorts; Figure 2E). The favourable survival for PAPPA2‐Mut was also verified after considering confounding factors (Table 1, multivariable analysis, HR, 0.28 [95% CI, 0.14–0.53], *p* < 0.001). These results suggested that *PAPPA2* mutation was associated with the clinical benefit of immunotherapy.

**TABLE 1:** Univariable and multivariable analyses of PFS in the NSCLC set

Characteristic	Univariable			Multivariable		
	HR	95% CI	*p* value	HR	95% CI	*p* value
Cohort						
Hellmann	‐	‐		‐	‐	
Miao	1.28	0.85, 1.93	0.2			
Rizvi	1.08	0.65, 1.78	0.8			
Gender						
Female	‐	‐		‐	‐	
Male	1.26	0.87, 1.82	0.2			
Age						
<65	‐	‐		‐	‐	
≥65	0.91	0.61, 1.35	0.6			
Unknown	1.58	0.89, 2.82	0.12			
Smoking						
Never	‐	‐		‐	‐	
Current	0.69	0.38, 1.24	0.2	1.07	0.57, 2.00	0.8
Former	0.58	0.37, 0.92	0.019	0.83	0.52, 1.33	0.4
Histology						
Non‐squamous	‐	‐		‐	‐	
Squamous	0.92	0.55, 1.54	0.7			
NSCLC NOS	0.36	0.05, 2.56	0.3			
Treatment						
Anti‐PD‐(L)1	‐	‐		‐	‐	
Anti‐PD‐(L)1 + Anti‐CTLA4	0.83	0.57, 1.21	0.3			
Line						
First	‐	‐		‐	‐	
Second or subsequent	1.16	0.66, 2.02	0.6			
Unknown	1.29	0.86, 1.93	0.2			
PDL1						
<1%	‐	‐		‐	‐	
1%–49%	0.91	0.53, 1.56	0.7	0.85	0.48, 1.50	0.6
≥50%	0.38	0.17, 0.86	0.02	0.38	0.16, 0.85	0.019
Unknown	0.98	0.59, 1.63	>0.9	0.85	0.51, 1.43	0.5
TMB						
Others	‐	‐		‐	‐	
Top 25%	0.35	0.22, 0.58	<0.001	0.47	0.27, 0.80	0.005
*PAPPA2*						
WT	‐	‐		‐	‐	
Mut	0.28	0.14, 0.53	<0.001	0.37	0.18, 0.78	0.009

Abbreviations: CI, confidence interval; HR, hazard ratio; NSCLC, non‐small cell lung cancer; PFS, progress free survival; TMB, tumour mutational burden.

Factors of TMB (HR, 0.35 [95% CI, 0.22–0.58]; *p* < 0.001; Table 1) and PD‐L1 (≥50%; HR, 0.38 [95% CI, 0.17–0.86]; *p* = 0.02; Table 1) were also significantly associated with superior PFS. In 100 patients with known PD‐L1 status, 35.3% and 16.9% had PD‐L1 ≥50% in patients with PAPPA2‐Mut and PAPPA2‐WT, respectively (Figure S1A). Patients with positive PD‐L1 expression (≥1%) and PAPPA2‐Mut tended to have a superior PFS (HR, 0.11 [95% CI, 0.02–0.46]; *p* = 0.003; Figure S1B).

### 3.2 Association between *PAPPA2* mutation and clinical benefits of ICIs therapy in the SKCM set

**FIGURE 3:**
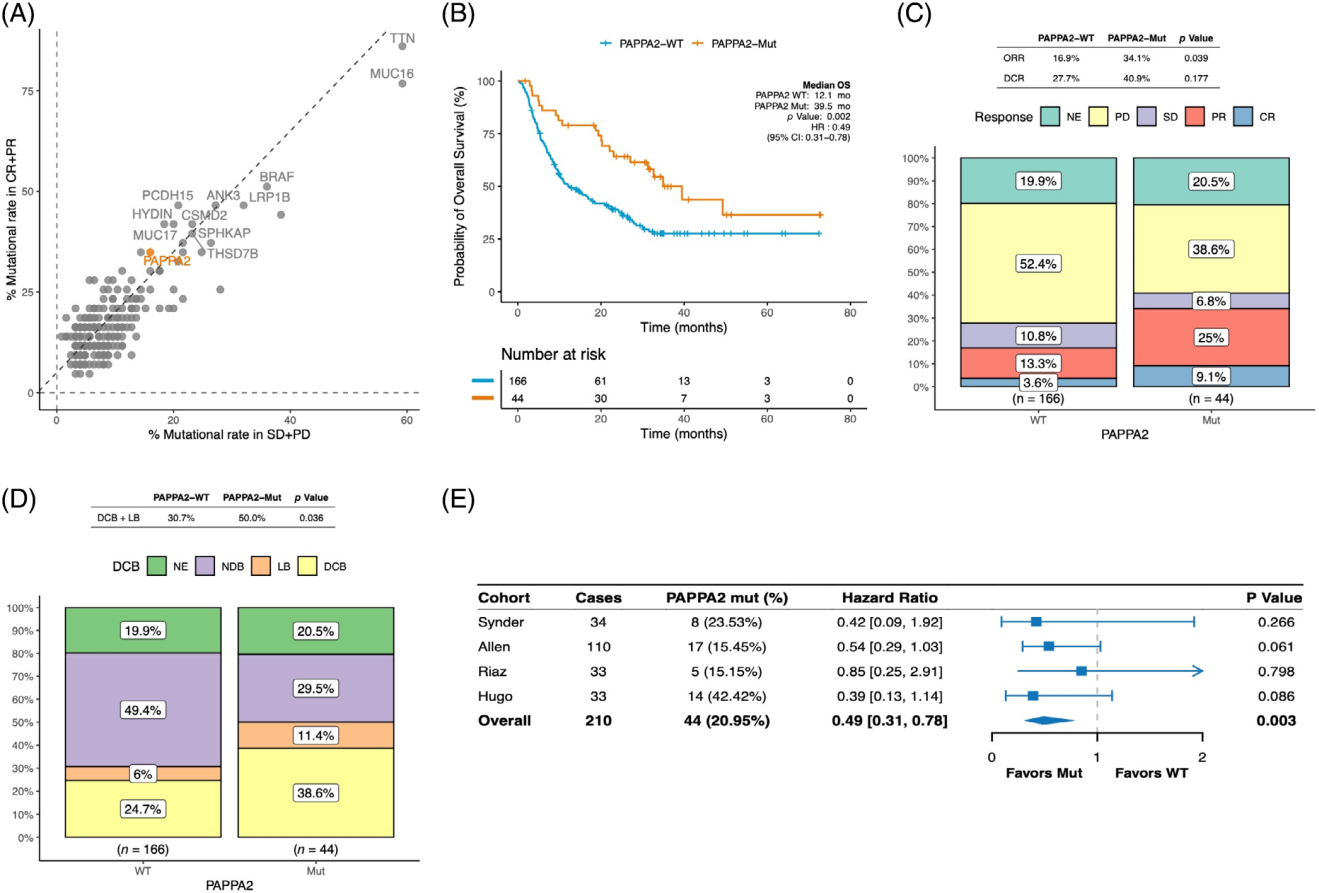
Association between *PAPPA2* mutation and clinical benefits of ICIs therapy in the SKCM set. (A) A scatter diagram displaying the mutational rate of different ICIs‐related gene mutations in patients with objective response (CR + PR) versus without (SD + PD) in the SKCM set, with *PAPPA2* mutation(34.9% vs. 16%) highlighted in orange. (B) Longer OS observed in the PAPPA2‐Mut group compared to the PAPPA2‐MT group in the SKCM set. (C) The response data on ORR and DCR of patients evaluated in the SKCM set. (D) The response data on DCB + LB of patients evaluated in the SKCM set. (E) The trend of prolonged OS in the PAPPA2‐Mut group consistently observed among four cohorts included in the SKCM set. DCB, durable clinical benefit; DCR, disease control rate; ICIs, immune checkpoint inhibitors; ORR, objective response rate; PFS, progress free survival; SKCM, skin cutaneous melanoma

In the SKCM set, *PAPPA2* mutation was also discovered to be enriched in patients with objective response (34.9%) versus without (16%; Figure 3A). We further validated the association between *PAPPA2* mutation and clinical benefits. Expectedly, patients with SKCM harbouring *PAPPA2* mutation had superior OS (HR, 0.49 [95% CI: 0.31–0.78]; *p* = 0.002; Figure 3B), higher ORR (34.1% vs. 16.9%; *p* = 0.039; Figure 3C) and higher DCB + LB (50.0% vs. 30.7%; *p* = 0.036; Figure 3D) compared with those with PAPPA2‐WT group. The prolonged OS in *PAPPA2* mutation patients with SKCM was consistently observed across four datasets included in the validation set (Figure 3E). The prolonged OS in PAPPA2‐Mut patients was also consistently observed in multivariable analysis (HR, 0.54 [95% CI, 0.32–0.91]; *p* = 0.021; Table 2). These results suggested that *PAPPA2* mutation was associated with the clinical benefit of immunotherapy.

**TABLE 2:** Univariable and multivariable analyses of overall survival in the SKCM set

Characteristic	Univariable			Multivariable		
	HR	95% CI	*p* value	HR	95% CI	*p* value
Cohort						
Riaz	‐	‐				
Synder	0.51	0.25, 1.03	0.06			
Allen	1.58	0.97, 2.58	0.067			
Hugo	0.75	0.39, 1.45	0.4			
Gender						
Female	‐	‐				
Male	0.87	0.58, 1.29	0.5			
Unknown	0.8	0.46, 1.39	0.4			
Age						
<65	‐	‐				
≥65	1.1	0.76, 1.59	0.6			
Unknown	0.92	0.56, 1.54	0.8			
M class						
M0	‐	‐		‐	‐	
M1a	1.72	0.55, 5.40	0.4	1.81	0.58, 5.69	0.3
M1b	2.39	0.81, 7.08	0.11	2.6	0.88, 7.69	0.084
M1c	3.71	1.36, 10.1	0.01	4.39	1.61, 12.0	0.004
Unknown	1.57	0.39, 6.27	0.5	1.53	0.38, 6.14	0.5
Treatment						
Anti‐CTLA‐4	‐	‐				
Anti‐PD‐1	0.71	0.48, 1.04	0.075			
Line						
First	‐	‐				
Second	0.62	0.34, 1.15	0.13			
Third or subsequent	0	0.00, Inf	>0.9			
Unknown	0	0.00, Inf	>0.9			
TMB						
Others	‐	‐		‐	‐	
Top 25%	0.56	0.36, 0.87	0.01	0.62	0.38, 1.02	0.06
*PAPPA2*						
WT	‐	‐		‐	‐	
Mut	0.49	0.31, 0.78	0.003	0.54	0.32, 0.91	0.021

Abbreviations: CI, confidence interval; HR, hazard ratio; SKCM, skin cutaneous melanoma; TMB, tumour mutational burden.

### 3.3 The association between *PAPPA2* mutation and TMB

**FIGURE 4:**
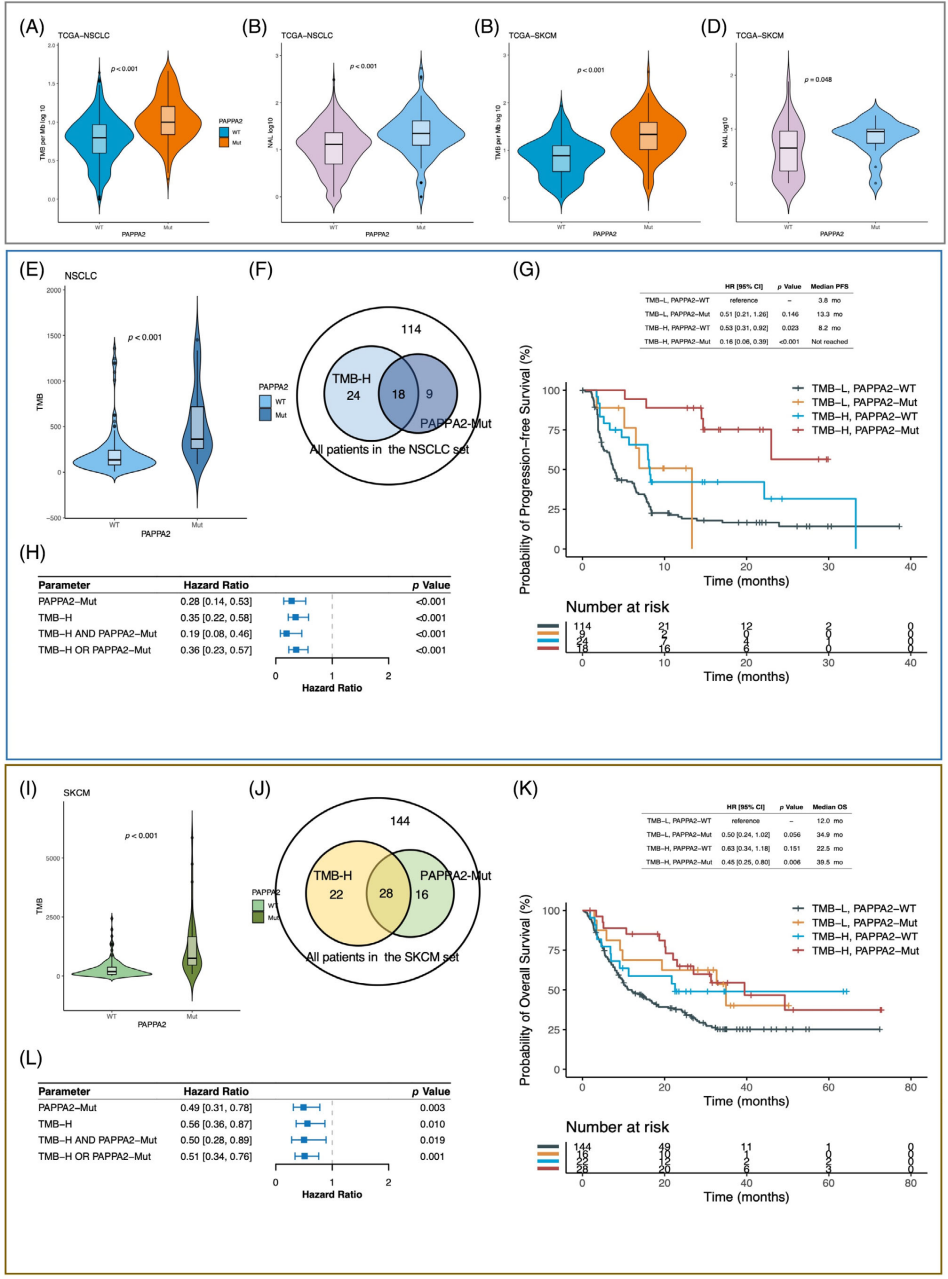
The association between *PAPPA2* mutation and TMB. (A–D) A comparison of TMB and NAL between PAPPA2‐Mut and PAPPA2‐WT groups in NSCLC and SKCM based on the TCGA database. (E) A comparison of TMB between PAPPA2‐Mut and PAPPA2‐WT groups in the NSCLC set. (F) A Venn diagram showing the concomitant presence of TMB‐H and PAPPA2‐Mut in the NSCLC set. (G) The HRs and *p* values of parameters including TMB‐H, *PAPPA2‐Mut*, TMB‐H and *PAPPA2‐Mu*t, and TMB‐H or *PAPPA2‐Mut* in the NSCLC set. (H) Kaplan–Meier curves comparing PFS among TMB‐H and PAPPA2‐Mut, TMB‐H and PAPPA2‐WT, TMB‐L and PAPPA2‐Mut, with TMB‐L and PAPPA2‐WT as a reference. (I) A comparison of TMB between PAPPA2‐Mut and PAPPA2‐WT groups in the SKCM set. (J) A Venn diagram showing the concomitant presence of TMB‐H and PAPPA2‐Mut in the SKCM set. (K) The HRs and *p* values of parameters including TMB‐H, *PAPPA2‐Mut*, TMB‐H and *PAPPA2‐Mut*, TMB‐H or *PAPPA2‐Mut*. (L) **T**he Kaplan–Meier curves comparing OS among TMB‐H & PAPPA2‐Mut, TMB‐H & PAPPA2‐WT, TMB‐L & PAPPA2‐Mut with TMB‐L and PAPPA2‐WT as a reference. NSCLC, non‐small cell lung cancer; SKCM, skin cutaneous melanoma; TMB, tumour mutational burden

Based on the TCGA database, findings in the TCGA‐NSCLC dataset showed that the PAPPA2‐Mut group had higher TMB (*p* < 0.001) and NAL (*p* < 0.001) levels than the PAPPA2‐WT group (Figure 4A,B), consistent in the TCGA‐SKCM dataset (TMB, *p* < 0.001; NAL, *p* = 0.048; Figure 4C,D).

For the NSCLC set, the PAPPA2‐Mut group had significantly higher TMB (*p* < 0.001) than the PAPPA2‐WT group (Figure 4E). In addition, a significant longer PFS was observed in patients with TMB‐H and PAPPA2‐Mut compared to those with TMB‐L and PAPPA2‐WT (HR, 0.16 [95% CI, 0.06–0.39]; *p* < 0.001; Figure 4F–G). Among all patients in the NSCLC set, those with TMB‐H or PAPPA2‐Mut (53/165) achieved significantly longer PFS (HR, 0.36 [95% CI, 0.23–0.57]; *p* < 0.001; Figure 4H) than counterparts. Similarly, the TMB level was significantly higher in PAPPA2‐Mut tumours in the SKCM set (*p* < 0.001, Figure 4I). In addition, a significant longer PFS was observed in patients with TMB‐H and PAPPA2‐Mut compared to those with TMB‐L and PAPPA2‐WT (HR, 0.45 [95% CI, 0.25–0.80]; *p* = 0.006; Figure 4I–K). Among all patients in the SKCM set, those with TMB‐H or PAPPA2‐Mut (66/210) achieved significantly longer OS (HR, 0.51 [95% CI, 0.34–0.76]; *p* < 0.001; Figure 4L). Results above revealed that combined utilization of *PAPPA2* mutation and TMB could expand the identification of ICIs therapy response in both NSCLC and SKCM patients.

### 3.4 Association between *PAPPA2* mutation and clinical benefits of ICIs in China cohort

In China cohort, which comprised 41 Chinese patients with NSCLC, we further investigated the association between *PAPPA2* mutation and clinical benefits. The trend of prolonged PFS in PAPPA2‐Mut was observed (HR, 0.28 [95% CI, 0.07–1.16]; *p* = 0.059; Figure S2A). Meanwhile, patients with *PAPPA2* mutation had a higher ORR (66.7% vs. 13.2%; *p* = 0.070; Figure S2B) and DCB (66.7% vs. 22.1%; *p* = 0.142; Figure S2C) compared with those in the PAPPA2‐WT group. Additionally, the TMB level in PAPPA2‐Mut was significantly higher compared to that in PAPPA2‐WT (*p* = 0.015, Figure S2D). Table S6 summarized the clinical characteristics of patients in China cohort.

### 3.5 Comparison to known predictive gene mutations of ICIs benefit

Among *PAPPA2* and other established predictive gene mutations, mutations of *PAPPA2*, *EPHA* family, *MUC16*, *LRP1B* and *TTN* brought superior prediction of ICIs benefit in both sets by univariable analysis (Table 3). PAPPA2‐Mut presented the lowest risk of progression or death in both sets (NSCLC: HR, 0.28 [95% CI, 0.04–0.53]; *p* < 0.001; SKCM set: HR, 0.49 [95% CI, 0.31–0.78]; *p* = 0.003). These results confirmed the remarkable prediction value of *PAPPA2* mutation in ICIs benefit.

**TABLE 3:** Compared with other known predictive gene mutations with univariable Cox analysis

Gene	NSCLC			SKCM		
	Mutation (%)	Hazard ratio	*p* value	Mutation (%)	Hazard ratio	*p* value
*ARID1A*	6.67	0.53 [0.22, 1.30]	0.167	6.67	0.69 [0.34, 1.42]	0.313
*CDKN2A*	6.67	0.38 [0.14, 1.04]	0.060	5.08	0.65 [0.27, 1.60]	0.348
*EGFR*	15.76	2.03 [1.28, 3.23]	0.003	5.08	0.84 [0.39, 1.81]	0.656
*EPHA_*family	19.39	0.46 [0.28, 0.78]	0.004	39.05	0.54 [0.37, 0.78]	0.001
*KEAP1*	16.97	0.65 [0.37, 1.13]	0.126	1.82	0.73 [0.18, 3.01]	0.662
*KMT2_*family	15.76	0.56 [0.32, 0.98]	0.041	28.57	0.68 [0.46, 1.01]	0.058
*KRAS*	28.48	0.61 [0.40, 0.94]	0.026	3.47	0.46 [0.11, 1.88]	0.281
*LRP1B*	26.06	0.53 [0.33, 0.84]	0.007	36.19	0.55 [0.37, 0.80]	0.002
*MUC16*	35.15	0.53 [0.35, 0.79]	0.002	64.29	0.63 [0.44, 0.89]	0.009
*NOTCH_*family	10.30	0.82 [0.45, 1.50]	0.521	24.29	0.66 [0.44, 1.01]	0.053
*PAPPA2*	16.36	0.28 [0.14, 0.53]	<0.001	20.95	0.49 [0.31, 0.78]	0.003
*POLD1*	7.34	0.50 [0.18, 1.36]	0.174	3.33	0.58 [0.21, 1.56]	0.279
*POLE*	1.82	0.00 [0.00, Inf]	0.995	3.95	0.63 [0.23, 1.72]	0.368
*STK11*	12.12	1.67 [0.96, 2.88]	0.068	0.70	2.40 [0.33, 17.42]	0.385
*TP53*	52.73	0.73 [0.50, 1.05]	0.092	11.43	1.58 [0.95, 2.64]	0.077
*TTN*	44.85	0.43 [0.29, 0.63]	<0.001	66.67	0.66 [0.46, 0.94]	0.021

Abbreviations: NSCLC, non‐small cell lung cancer; SKCM, skin cutaneous melanoma.

### 3.6 *PAPPA2* mutation was not a prognostic factor

To evaluate whether the survival benefit of ICIs therapy in patients with PAPPA2‐Mut was simply resulted from the general prognostic impact of *PAPPA2* mutation, we further assessed the PFS and OS difference between PAPPA2‐Mut and PAPPA2‐WT patients with NSCLC or SKCM in TCGA database (Figure S3). Obviously, there was no PFS or OS difference owing to *PAPPA2* mutation in lung adenocarcinoma (LUAD), lung squamous carcinoma (LUSC), NSCLC or SKCM. Therefore, *PAPPA2* mutation may be a predictive but not a prognostic factor in ICIs treatment for patients with NSCLC as well as SKCM.

### 3.7 Potential mechanisms associated with *PAPPA2* mutation in anti‐tumour immunity

**FIGURE 5:**
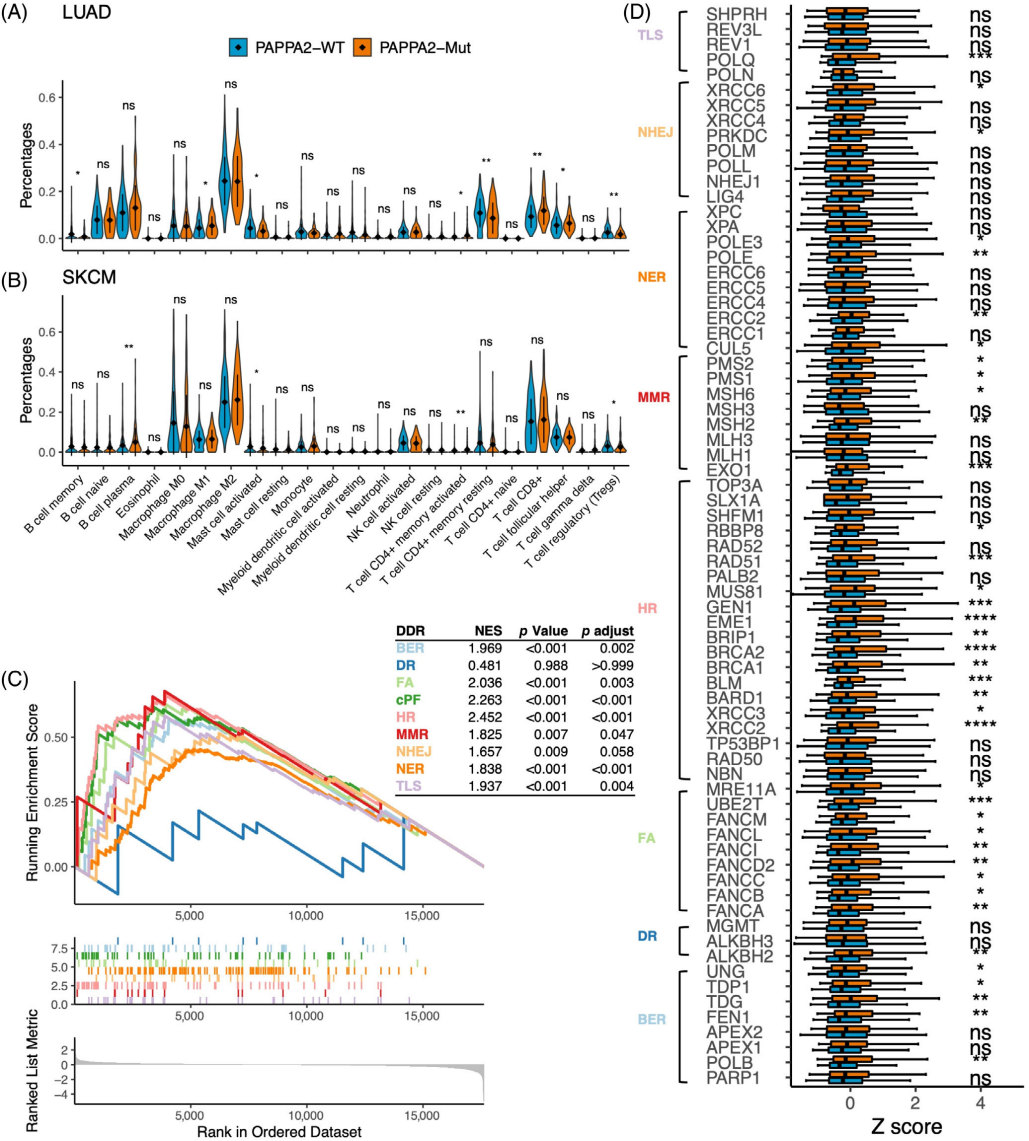
*PAPPA2* mutation was associated with enhanced anti‐tumour immunity in the TCGA database. (A,B) Violin plots depicting the infiltration of immune cells in PAPPA2‐Mut tumours and PAPPA2‐WT tumours in TCGA‐LUAD and TCGA‐SKCM. (C) The enrichment of DDR pathways between PAPPA2‐Mut and PAPPA2‐WT groups in TCGA‐LUAD. (D) Box plots comparing the expression of DDR‐related genes between PAPPA2‐Mut and PAPPA2‐WT groups in TCGA‐LUAD (*p < 0.05, **p < 0.01, ***p < 0.001)

To investigate the potential mechanisms associated with *PAPPA2* mutation, we used the CIBERSORT algorithm to estimate the immune cell infiltration status based on the TCGA database. A comparing analysis in both TCGA‐LUAD and TCGA‐SKCM datasets showed that the PAPPA2‐Mut group had revealed higher activated CD4 memory T cells and lower Treg cells than PAPPA2‐WT tumours (Figure 5A,B).

DDR signalling pathways and related genes based on RNA‐Seq data from the TCGA database were analysed. GSEA of Reactome revealed that gene sets related to the DDR pathways (the nonhomologous end‐joining (NHEJ) pathway, homologous recombination repair (HR) pathway, etc.) were significantly enriched in PAPPA2‐Mut tumours (*p* < 0.001, Figure 5C) in TCGA‐LUAD. Moreover, TCGA‐LUAD tumours with *PAPPA2* mutation had increased mRNA expression of DDR‐related genes (Figure 5D).

## 4 DISCUSSION

The application of advanced technologies and bioinformatic tools has enabled us to investigate the role of certain factors in various diseases.^41^, ^42^ Likewise, in this study for preliminary analysis, we observed an enrichment of *PAPPA2* mutation in NSCLC and SKCM, as well as a discrepancy in patients with objective response versus without. In addition, independent of PD‐L1 expression or TMB status, *PAPPA2* mutation displayed a strong association with better clinical outcomes in patients with NSCLC and SKCM after receiving ICIs therapy. However, the correlation between *PAPPA2* mutation and prognosis has not drawn much attention even though Ayako Suzuki et al identified *PAPPA2* mutation as prolonged‐prognosis‐related gene of LUAD in 2013.^25^ So far as we know, this is the first study to elucidate the role of *PAPPA2* mutation in stratifying the efficacy of ICIs therapy.

Currently, PD‐L1 expression and TMB status were the most utilized predictive biomarkers of ICIs. However, the prediction value could be affected by various factors, such as different cut‐off points, calculating algorithms and detecting assays, etc.^43^, ^44^ Hence, limitations remained in practice due to inconsistency and heterogeneity.^12^, ^45^ Our results revealed the improved survival in patients with *PAPPA2* mutation, suggesting *PAPPA2* mutation was a potential predictive biomarker of ICIs, complementing to PD‐L1 and TMB. As a single gene biomarker, the qualitative detection of *PAPPA2* mutation made it objective to identify potential beneficiaries. Noteworthy, a comparison of *PAPPA2* mutation with other published ICIs‐related gene mutations showed a prominently potential predicting ability, such as *EPHA* family, *MUC16*, *LRP1B* and *TTN*, etc.^16^, ^18^, ^46^, ^47^ However, it also revealed the instability of single genes to some extent. Intriguingly, patients revealed the prediction instability or TMB‐H demonstrated a beneficial significance in both sets, suggesting a combination of *PAPPA2* mutation and TMB could expand the identification of potential responders to ICIs therapy.

We utilized a multidimensional TCGA database to explore how PAPPA2‐Mut tumours respond to immunotherapy. PAPPA2‐Mut tumours were discovered with higher levels of TMB and NAL, which are associated with increased tumour immunogenicity. Then RNA‐Seq data revealed that *PAPPA2* mutation was significantly associated with higher activated CD4 memory T cells and lower Treg cells, suggesting an enhanced anti‐tumour immunity.^48^, ^49^ Moreover, PAPPA2‐Mut tumours were enriched with multiple DDR pathways, which are associated with the efficacy of ICIs treatment in tumours.^50^ Alterations of DDR‐related genes are closely correlated with higher TMB.^51^ Hence, PAPPA2‐Mut tumours were correlated with increased immunogenicity and enhanced anti‐tumour immunity, implying a better performance in ICIs therapy.

Our study has several limitations. First, to our knowledge, *PAPPA2* mutation has not been added to existing targeted gene panels. The retrospective study design and limited public data might introduce information bias. To minimize it, we extracted common clinical characteristics across data sets for NSCLC and SKCM respectively, and unified the category definition and processing algorithms. We also conducted multivariable models and consistent results were found. Secondly, interestingly enough, in the TCGA database, we did not find the PFS/OS difference between PAPPA2‐Mut and PAPPA2‐WT neither in LUAD, LUSC, NSCLC or SKCM, suggesting it was not a prognosis biomarker, contrary to its role in Ayako Suzuki cohort (7/90, about 7.7%) as a prognosis biomarker.^25^ In addition, though varying among cohorts, *PAPPA2* mutation rates in the NSCLC set (14.67%–17.86%) are much higher than Asian cohorts, like Ayako Suzuki et al cohort (Japanese, 7.7%) and China cohort (Chinese, 7.1%). It seems that *PAPPA2* mutation rates and corresponding functions vary by race. Hence, several parameters may contribute to the insignificance of *PAPPA2* mutation in this relatively small‐sized China cohort, though the tendency supported the prediction value of *PAPPA2* mutation to some extent. All in all, larger prospective clinical trials with multidimensional data and mechanism‐exploring experiments are needed to clarify and validate the predictive capacity and functional alterations of *PAPPA2* mutation.

## 5 CONCLUSION

In summary, our study explored the association between *PAPPA2* mutation and the clinical benefit of ICIs therapy in NSCLC and SKCM. Our results demonstrated that patients with *PAPPA2* mutation were associated with better clinical outcomes in ICIs treatment via activated immunogenicity and enhanced anti‐tumour immunity. Thus, *PAPPA2* mutation could act as a potential predictive biomarker for ICIs therapy in NSCLC and SKCM, warranting further prospective studies.

## AUTHOR CONTRIBUTIONS

Zhijie Wang and Jie Wang developed the concept and designed the study. Lele Zhao, Jianchun Duan, Qin Zhang and Hua Bai performed the statistical analysis. Yiting Dong, Yangyang Yu, Lele Zhao, Dongsheng Chen and Si Li wrote the manuscript. Mingzhe Xiao, Qianqian Duan, Jie Wang, Zhijie Wang, Tingting Sun and Chuang Qi performed a critical revision of the manuscript for the intellectual content. All authors read and approved the final manuscript.

## FUNDING INFORMATION

Support for the study was provided by CAMS Innovation Fund for Medical Sciences (No. 2021‐1‐I2M‐012 to Dr Zhijie Wang); CAMS Key lab of translational research on lung cancer (No. 2018PT31035 to Dr Jie Wang), the National Natural Sciences Foundation (Nos. 81871889 and 82072586 to Dr Zhijie Wang) and Beijing Natural Science Foundation (No. 7212084 to Dr Zhijie Wang).

## CONFLICT OF INTEREST

Lele Zhao, Dongsheng Chen, Si Li, Yangyang Yu, Mingzhe Xiao, Qin Zhang, Qianqian Duan, Tingting Sun and Chuang Qi were employed by Jiangsu Simcere Diagnostics Co., Ltd. The remaining authors declare that the research was conducted in the absence of any commercial or financial relationships that could be construed as a potential conflict of interest.

## Supporting information


**Appendix S1** Supporting information.

## Data Availability

The public cohorts used in this study were publicly available as described in the ‘Materials and methods’ section. The China cohort is available from the corresponding author on reasonable request.
