# Supplementary material for: PAPPA2 mutation as a novel indicator stratifying beneficiaries of immune checkpoint inhibitors in skin cutaneous melanoma and non‐small cell lung cancer
Source: Cell Prolif. 2022 Jul 10;55(9):e13283. doi: 10.1111/cpr.13283 (PMC9436912; doi:10.1111/cpr.13283)
Supplement: Supplementary file 1 — Appendix S1 Supporting information. [file CPR-55-e13283-s001.docx]

**Supplementary Materials**

1. **Figure legends of Supplementary Figures S1-3**
2. **Supplementary Table S1**
3. **Supplementary Table S2**
4. **Supplementary Table S3**
5. **Supplementary Table S4**
6. **Supplementary Table S5**
7. **Supplementary Table S6**

**Figure legends of Supplementary Figures**

**
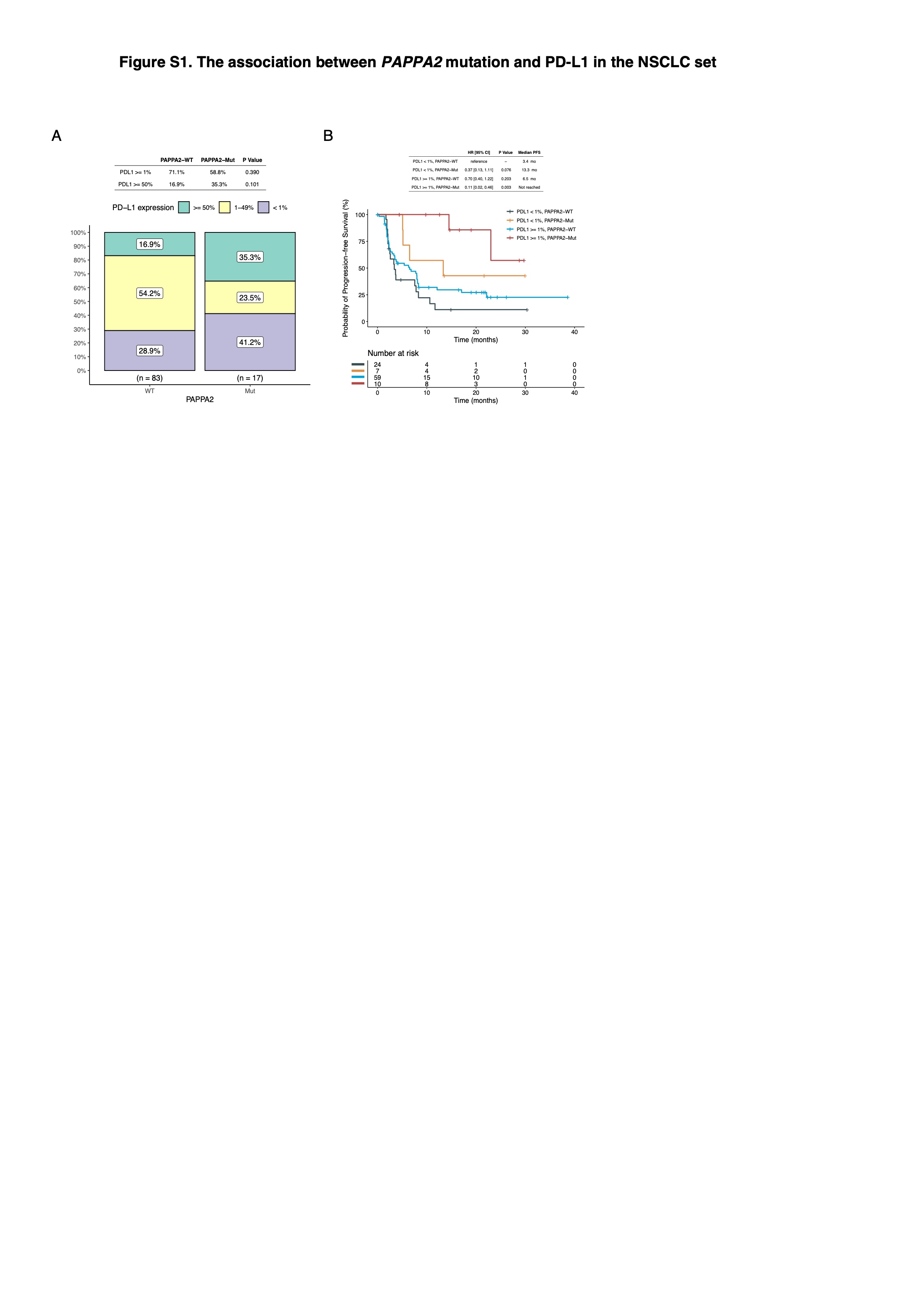
**

**Supplementary Figure S1. The association between PAPPA2 Mutation and PD-L1 in the NSCLC set**

**A.** The ratio of patients with distinct PD-L1 expression levels (≥ 50%, 1-49% and < 1%) in PAPPA2-Mut and PAPPA2-WT groups.

**B.** The Kaplan-Meier curves comparing PFS among PD-L1 ≥ 1% & PAPPA2-Mut, PD-L1 ≥ 1% & PAPPA2-WT, PD-L1 < 1% & PAPPA2-Mut and PD-L1 < 1% & PAPPA2-WT groups.

**
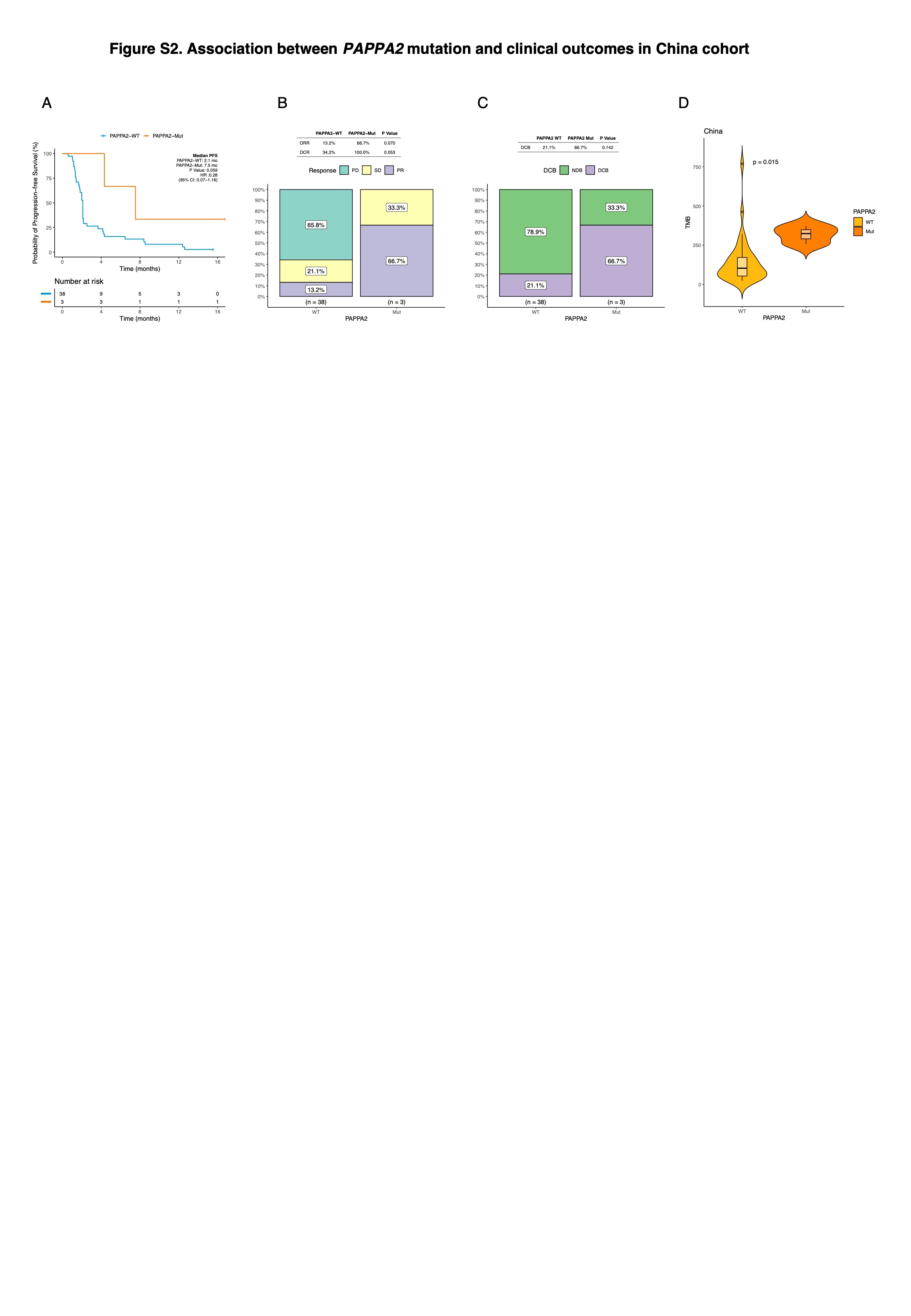
**

**Supplementary Figure S2. Association between PAPPA2 mutation and clinical outcomes in China cohort**

**A**. Longer PFS was observed in the PAPPA2-Mut group compared to the PAPPA2-WT group in China cohort.

**B.** The response data on ORR and DCR of patients were evaluated in China cohort.

**C.** The response data on DCB of patients were evaluated in China cohort.

**D.** Comparison of TMB and NAL between PAPPA2-Mut and PAPPA2-WT groups.

**
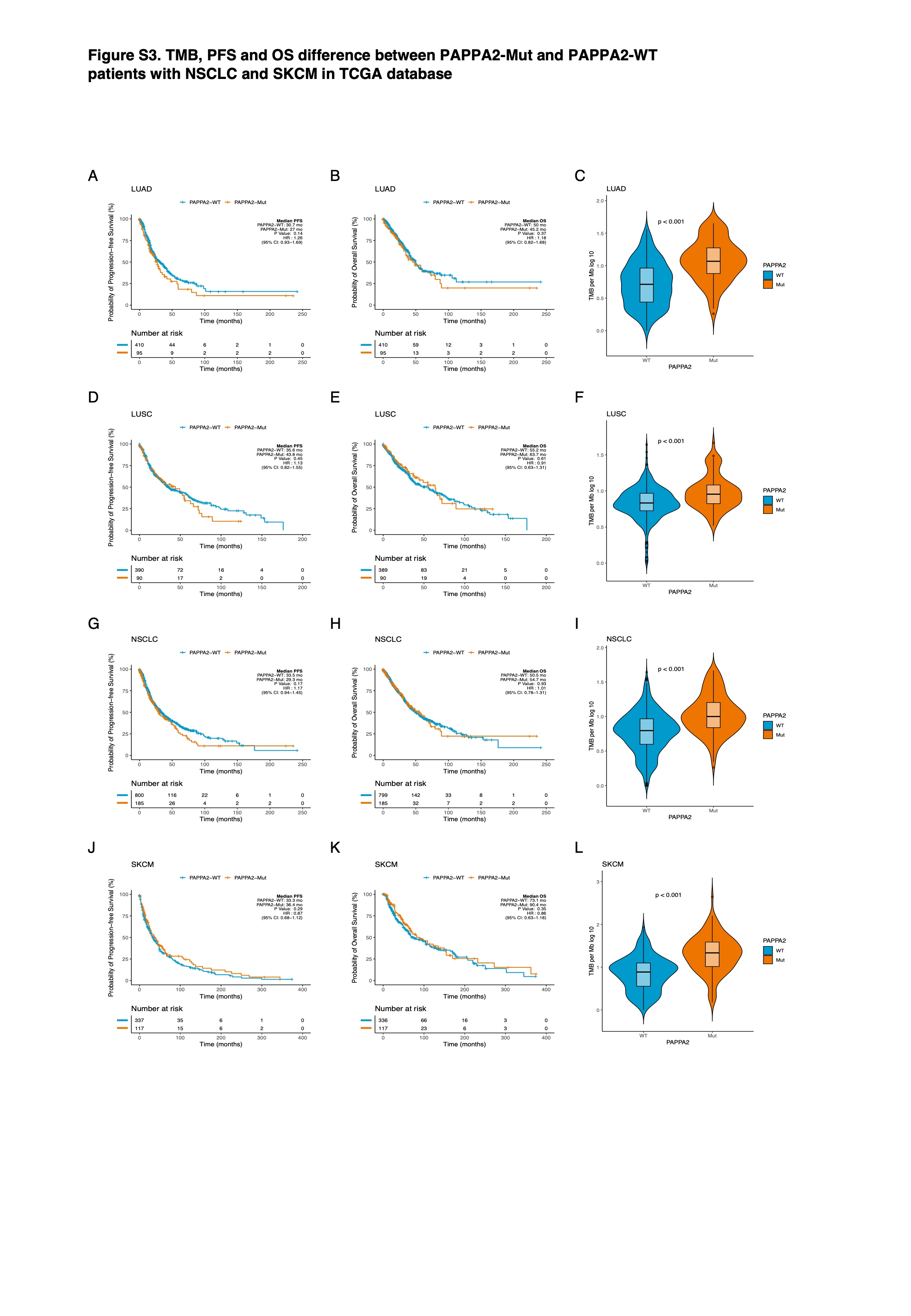
**

**Supplementary Figure S3. TMB, PFS and OS difference between PAPPA2-Mut and PAPPA2-WT patients with NSCLC and SKCM in TCGA database**

Survival between *PAPPA2* mutation status for LUAD (**A-B**), lung squamous carcinoma (LUSC) (**D-E**), NSCLC (**G-H**), and SKCM (**J-K**) cohort. Kaplan-Meier survival plots, P value of log-rank test are shown. Comparison of TMB between PAPPA2-Mut and PAPPA2-WT group in LUAD (**C**), LUSC (**F**), NSCLC (**I**), and SKCM (**L**) cohort. Statistics based on two-tailed Mann-Whitney *U* test

**Supplementary Table S1. Clinical stratification of immune cohorts**

| **Cohort** | **Durable Clinical Benefit (DCB)** | **No Durable Benefit (NDB)** | **Other** | **Source** |
| --- | --- | --- | --- | --- |
| **NSCLC** | CR/PR  SD with PFS > 6 months | PD  SD with PFS < 6 months | NA | Roh et al. 2017 |
| **SKCM** | CR/PR  SD with OS > 12 months | PD  SD with OS < 12 months | Long-term survival (LB):  PD with OS > 2 years | Van Allen et al. 2015 |

**Supplementary Table S2. DDR core genes**

| **Pathway** | **Gene** |
| --- | --- |
| **BER** | *PARP1, POLB, APEX1, APEX2, FEN1, TDG, TDP1, UNG* |
| **FA** | *FANCA, FANCB, FANCC, FANCD2, FANCI, FANCL, FANCM, UBE2T* |
| **HR** | *MRE11A, NBN, RAD50, TP53BP1, XRCC2, XRCC3, BARD1, BLM, BRCA1, BRCA2, BRIP1, EME1, GEN1, MUS81, PALB2, RAD51, RAD52, RBBP8, SHFM1, SLX1A, TOP3A* |
| **MMR** | *EXO1, MLH1, MLH3, MSH2, MSH3, MSH6, PMS1, PMS2* |
| **NER** | *CUL5, ERCC1, ERCC2, ERCC4, ERCC5, ERCC6, POLE, POLE3, XPA, XPC* |
| **NHEJ** | *LIG4, NHEJ1, POLL, POLM, PRKDC, XRCC4, XRCC5, XRCC6* |
| **TLS** | *POLN, POLQ, REV1, REV3L, SHPRH* |
| **DR** | *ALKBH2, ALKBH3, MGMT* |

**Supplementary Table S3. DDR genesets**

| **ID** | **Abbreviation** | **Description** |
| --- | --- | --- |
| R-HSA-110313 | TLS | Translesion synthesis by Y family DNA polymerases bypasses lesions on DNA template |
| R-HSA-5358508 | MMR | Mismatch Repair |
| R-HSA-5685942 | HR | HDR through Homologous Recombination (HRR) |
| R-HSA-5693571 | NHEJ | Nonhomologous End-Joining (NHEJ) |
| R-HSA-5696398 | NER | Nucleotide Excision Repair |
| R-HSA-6783310 | FA | Fanconi Anemia Pathway |
| R-HSA-69473 | cPF | G2/M DNA damage checkpoint |
| R-HSA-73884 | BER | Base Excision Repair |
| R-HSA-73942 | DR | DNA Damage Reversal |

**Supplementary Table S4. Clinical characteristics of patients in the NSCLC set**

| **Variable** | **N** | **PAPPA2-WT, N = 138** | **PAPPA2-Mut, N = 27** | **P Value^1^** |
| --- | --- | --- | --- | --- |
| **Age** | 150 |  |  | 0.072 |
| Mean (SD) |  | 63 (10) | 60 (8) |  |
| Unknown |  | 12 | 3 |  |
| **Gender** | 165 |  |  | 0.13 |
| Female |  | 70 (51%) | 18 (67%) |  |
| Male |  | 68 (49%) | 9 (33%) |  |
| **Cohort** | 165 |  |  | 0.9 |
| Hellmann |  | 64 (46%) | 11 (41%) |  |
| Miao |  | 46 (33%) | 10 (37%) |  |
| Rizvi |  | 28 (20%) | 6 (22%) |  |
| **Smoking** | 164 |  |  | 0.037 |
| Never |  | 33 (24%) | 1 (3.8%) |  |
| Current |  | 23 (17%) | 7 (27%) |  |
| Former |  | 82 (59%) | 18 (69%) |  |
| Unknown |  | 0 | 1 |  |
| **Histology** | 165 |  |  | 0.13 |
| Non-squamous |  | 109 (79%) | 26 (96%) |  |
| Squamous |  | 26 (19%) | 1 (3.7%) |  |
| NSCLC NOS |  | 3 (2.2%) | 0 (0%) |  |
| **Treatment** | 165 |  |  | 0.6 |
| Anti-PD-(L)1 |  | 74 (54%) | 16 (59%) |  |
| Anti-PD-(L)1 + Anti-CTLA4 |  | 64 (46%) | 11 (41%) |  |
| **Line** | 165 |  |  | 0.7 |
| First |  | 72 (52%) | 12 (44%) |  |
| Second or subsequent |  | 20 (14%) | 5 (19%) |  |
| Unknown |  | 46 (33%) | 10 (37%) |  |
| **PDL1** | 165 |  |  | 0.1 |
| < 1% |  | 24 (17%) | 7 (26%) |  |
| 1-49% |  | 45 (33%) | 4 (15%) |  |
| ≥ 50% |  | 14 (10%) | 6 (22%) |  |
| Unknown |  | 55 (40%) | 10 (37%) |  |
| **TMB** | 165 |  |  | <0.001 |
| Median (IQR) |  | 136 (80, 240) | 363 (260, 719) |  |
| ^1^Wilcoxon rank sum test; Pearson's Chi-squared test; Fisher's exact test | | | | |

**Supplementary Table S5. Patient characteristics in the SKCM set**

| **Variable** | **N** | **PAPPA2-WT, N = 166** | **PAPPA2-Mut, N = 44** | **P Value^1^** |
| --- | --- | --- | --- | --- |
| **Cohort** | 210 |  |  | 0.007 |
| Riaz |  | 28 (17%) | 5 (11%) |  |
| Synder |  | 26 (16%) | 8 (18%) |  |
| Allen |  | 93 (56%) | 17 (39%) |  |
| Hugo |  | 19 (11%) | 14 (32%) |  |
| **Age** | 177 |  |  | 0.048 |
| Mean (SD) |  | 58 (15) | 63 (14) |  |
| Unknown |  | 28 | 5 |  |
| **Gender** | 210 |  |  | 0.4 |
| Female |  | 39 (23%) | 14 (32%) |  |
| Male |  | 99 (60%) | 25 (57%) |  |
| Unknown |  | 28 (17%) | 5 (11%) |  |
| **Treatment** | 210 |  |  | 0.059 |
| anti-CTLA-4 |  | 119 (72%) | 25 (57%) |  |
| anti-PD-1 |  | 47 (28%) | 19 (43%) |  |
| **Line** | 210 |  |  | 0.3 |
| First |  | 142 (86%) | 37 (84%) |  |
| Second |  | 19 (11%) | 4 (9.1%) |  |
| Third or subsequent |  | 2 (1.2%) | 0 (0%) |  |
| Unknown |  | 3 (1.8%) | 3 (6.8%) |  |
| **M class** | 210 |  |  | >0.999 |
| M0 |  | 11 (6.6%) | 2 (4.5%) |  |
| M1a |  | 18 (11%) | 4 (9.1%) |  |
| M1b |  | 21 (13%) | 7 (16%) |  |
| M1c |  | 109 (66%) | 30 (68%) |  |
| Unknown |  | 7 (4.2%) | 1 (2.3%) |  |
| **TMB** | 210 |  |  | <0.001 |
| Median (IQR) |  | 190 (63, 373) | 750 (458, 1,661) |  |
| \| ^1^Wilcoxon rank sum test; Pearson's Chi-squared test; Fisher's exact test \| \| --- \| | | | | |

**Supplementary Table S6. Patient characteristics in China cohort**

| **Variable** | **PAPPA2-WT, N = 38** | **PAPPA2-Mut, N = 3** | **P value^1^** |
| --- | --- | --- | --- |
| **Age** |  |  | 0.5 |
| Mean (SD) | 54 (11) | 59 (6) |  |
| **Gender** |  |  | 0.3 |
| Female | 15 (39%) | 0 (0%) |  |
| Male | 23 (61%) | 3 (100%) |  |
| **Smoking** |  |  | 0.2 |
| Never | 19 (50%) | 0 (0%) |  |
| Former | 19 (50%) | 3 (100%) |  |
| **Histology** |  |  | >0.9 |
| Non-squamous | 21 (55%) | 2 (67%) |  |
| NSCLC NOS | 4 (11%) | 0 (0%) |  |
| Squamous | 13 (34%) | 1 (33%) |  |
| **Line** |  |  | 0.14 |
| First | 1 (2.6%) | 1 (33%) |  |
| Second or subsequent | 37 (97%) | 2 (67%) |  |
| **PDL1** |  |  | 0.2 |
| < 1% | 20 (53%) | 2 (67%) |  |
| 1-49% | 7 (18%) | 0 (0%) |  |
| ≥ 50% | 1 (2.6%) | 1 (33%) |  |
| Unknown | 10 (26%) | 0 (0%) |  |
| **TMB** |  |  | 0.015 |
| Median (IQR) | 104 (54, 172) | 324 (290, 348) |  |
| ^1^Wilcoxon rank sum test; Pearson's Chi-squared test; Fisher's exact test |  |  |  |
